# Editorial: Food salt reduction strategies and nutritional function evaluation

**DOI:** 10.3389/fnut.2024.1367071

**Published:** 2024-04-19

**Authors:** Tao Feng, Weitong Cai, Jun Lu, Honglei Tian

**Affiliations:** ^1^School of Perfume and Aroma Technology, Shanghai Institute of Technology, Shanghai, China; ^2^The University of Auckland, Auckland, New Zealand; ^3^School of Food Engineering and Nutrition Science, Shaanxi Normal University, Xi'an, China

**Keywords:** salt reduction, policy, investigation, education, WHO

Almost the entire population consumes too much sodium. The global average sodium intake for adults is 4,310 mg/day (equivalent to 10.78 g/day of salt) (1). That's more than double the World Health Organization's recommended daily sodium intake for adults of < 2,000 mg (equivalent to 5 grams of salt per day). The main health effects of a high-sodium diet are increased blood pressure and increased risk of cardiovascular disease, stomach cancer, obesity, osteoporosis, Meniere's disease and kidney disease. It is estimated that 1.89 million deaths each year are associated with consuming too much sodium (2). Reducing sodium intake is one of the most cost-effective measures to improve health and reduce the burden of non-communicable diseases: for every $1 invested in scaling up sodium intake interventions, there will be a return of at least $12. Therefore, a total of eight papers have been published in this subject.

Ming et al. reported that in people without high blood pressure, higher sodium intake was associated with an increased risk of diabetes, with a 1.20-fold increase in the risk of diabetes for every 1,000 mg increase in sodium intake. They provide clues to the causes of diabetes and further prospective studies are needed to provide recommendations for primary prevention of diabetes in the United States (Ming et al.). In Zhang, Sun et al.'s study, they focused on the development and preliminary implementation studies of the MHealth based School Health Education System (EduSaltS), which aims to scale up salt reduction in China. Finally, they found that EduSaltS was developed based on successfully tested interventions and appropriate scale-up frameworks (Zhang, Sun et al.). The best technology of ultrasonic desalting of Mianning ham was studied (He et al.). Through a series of studies, they found that the process significantly improved the desalting rate, texture and sensory quality of Mianning ham, providing solid theoretical support for the back-end desalting processing of ham (He et al.).

In Ethiopia, a study by Temech et al. focused on iodized salt adequacy and related factors in households in the Bahir Dar Zuria region of northwestern Ethiopia in 2022. They found that the proportion of households in the region with enough iodized salt (46.5%) is still very low and falls short of the national target level. Therefore, improving access to iodized salt at home is critical. In another study, the authors used a clinical trial to observe the effect of a low-sodium salt preparation combined with the Chinese modified DASH diet on lowering blood pressure in patients with hypertension and type 2 diabetes. Their preliminary results suggest that a low sodium salt concentration of 23 and 52% combined with the CM-DASH diet can effectively reduce sodium intake and increase potassium intake in patients with hypertension and type 2 diabetes, leading to “salt reduction” and the successful, integrated management of hypertension and type 2 diabetes (Zhang Z. et al.). Zhang, Zhang et al. conducted an exploratory study from a multinational research group. The study found that most stakeholders support setting sodium targets for different pre-packaged foods and should be implemented in conjunction with strategies to reduce the casual use of salt in Chinese cooking and eating processes. They also provide information on the perspectives, considerations, opportunities and challenges of an effective goal-setting policy in China (Zhang, Zhang et al.). A research team in Japan explored the economic impact of Japan's diet salt reduction policy on the prevention of cardiovascular disease through a simulation study of hypothetical scenarios. They found that forced reformulation at optimal cost may be economically superior to other alternatives in Japan (Ikeda et al.). A research team in Anhui Province examined the salt reduction behavior of adults in Anhui province in 2019 through a cross-sectional survey of 3,378 participants. They ultimately concluded that Anhui residents were not sufficiently knowledgeable about salt reduction. Age, sex, education level, high blood pressure and marital status are the main determinants. Their findings have significant implications for policy makers who want to develop salt reduction strategies (Xing et al.).

## Author contributions

TF: Writing—original draft, Writing—review & editing. WC: Writing—original draft. JL: Writing—review & editing, Conceptualization, Data curation, Formal analysis, Funding acquisition, Investigation, Methodology, Project administration, Resources, Software, Supervision, Validation, Visualization. HT: Conceptualization, Data curation, Formal analysis, Funding acquisition, Investigation, Methodology, Project administration, Resources, Software, Supervision, Validation, Visualization, Writing—review & editing.
